# Cost-Effectiveness of Pazopanib in Advanced Soft Tissue Sarcoma in the United Kingdom

**DOI:** 10.1155/2014/481071

**Published:** 2014-06-12

**Authors:** Jordan Amdahl, Stephanie C. Manson, Robert Isbell, Ayman Chit, Jose Diaz, Lily Lewis, Thomas E. Delea

**Affiliations:** ^1^Policy Analysis Inc. (PAI), Four Davis Court, Brookline, MA 02445, USA; ^2^GlaxoSmithKline, Stockley Park West, Uxbridge, Middlesex UB11 1BT, UK; ^3^GlaxoSmithKline, 7333 Mississauga Road, Mississauga, ON, Canada L5N 6L4; ^4^University of Toronto, 144 College Street, Toronto, ON, Canada M5S 3M2; ^5^York Health Economics Consortium, Level 2, Market Square, University of York, Heslington, York YO10 5NH, UK

## Abstract

In the phase III PALETTE trial, pazopanib improved progression-free survival (PFS) compared with placebo in patients with advanced/metastatic soft tissue sarcomas (mSTS) who had received prior chemotherapy. We used a multistate model to estimate expected PFS, overall survival (OS), lifetime STS treatment costs, and quality-adjusted life-years (QALYs) for patients receiving pazopanib, placebo, trabectedin, ifosfamide, or gemcitabine plus docetaxel as second-line mSTS therapies. The cost-effectiveness of pazopanib was expressed as the incremental costs per QALY gained. Estimates of PFS/OS, adverse events, and utilities for pazopanib and placebo were from the PALETTE trial. Estimates of relative effectiveness of the other comparators were from an unadjusted indirect comparison versus pazopanib. Costs were from published sources. Pazopanib is estimated to increase QALYs by 0.128 and costs by *£*7,976 versus placebo; cost per QALY gained with pazopanib versus placebo is estimated to be *£*62,000. Compared with the other chemotherapies, pazopanib provides similar QALYs at a lower cost. Pazopanib may not be cost-effective versus placebo but may be cost-effective versus the most commonly used active treatments, although this conclusion is uncertain. Given the unmet need for effective treatments for mSTS, pazopanib may be an appropriate alternative to some currently used medications in the United Kingdom.

## 1. Introduction

Soft tissue sarcomas (STS) are a rare group of solid tumors originating from mesenchymal cells and their precursors. STS includes more than 50 histological subtypes but accounts for less than 1% of all new malignancies in adults and approximately 2% of total cancer-related mortality [1, 2]. In 2010, 3,272 persons in the United Kingdom (UK) were diagnosed with cancer of connective and soft tissue [3].

Surgery, often combined with radiation therapy, offers the only potential cure for localized STS; however, approximately one-half of all patients with STS eventually develop local recurrence or metastases following surgery, radiotherapy, or both [4, 5]. Advanced STS is typically treated with palliative chemotherapy, and the median overall survival (OS) from the time metastases are found is 14 months [6].

Doxorubicin, alone or combined with ifosfamide, is the standard of care for first-line treatment of STS [5, 7, 8]. Although there is no standard of care following first-line chemotherapy, treatments recommended as second-line therapy by the British Sarcoma Group and the European Society for Medical Oncology are trabectedin, ifosfamide, gemcitabine plus docetaxel, taxanes (including docetaxel), gemcitabine, and dacarbazine [5, 7, 8]. Results from the Sarcoma Treatment and Burden of Illness in North America and Europe (SABINE) trial, a retrospective chart review of patients with metastatic STS, reported that gemcitabine plus docetaxel was the most frequently used second-line therapy, followed by ifosfamide monotherapy. Trabectedin was the most frequently used third-line therapy, followed by investigational drugs [9]. Current UK treatment guidelines recommend treatment with trabectedin, gemcitabine, gemcitabine plus docetaxel, or ifosfamide for patients who fail initial chemotherapy with doxorubicin-based therapy [5, 7].

Pazopanib (GW786034, Votrient, GlaxoSmithKline, Research Triangle Park, NC, USA), a multiple tyrosine kinase inhibitor, is approved for use in the United States, Canada, and the European Union for the treatment of advanced (unresectable and/or metastatic) STS (aSTS) in patients who have received prior chemotherapy [10–12]. Pazopanib was investigated in the PALETTE trial (ClinicalTrials.gov identifier: NCT00753688), a randomized, phase III investigation of pazopanib versus placebo in 369 patients with aSTS who had received prior chemotherapy [12]. Results of the PALETTE trial showed pazopanib improved progression-free survival (PFS) versus placebo (median 4.6 versus 1.6 months, resp.; hazard ratio [HR], 0.35; 95% confidence interval [CI], 0.26–0.48; *P* < 0.001) [10]. Median OS, a secondary objective of the trial, was 12.6 versus 10.7 months, respectively (HR, 0.87; 95% CI, 0.67–1.12; *P* = 0.26) [10]. However, posttreatment anticancer therapy (PTACT) was received by 62% in the placebo group compared with 45% in the pazopanib group at the data cutoff date [12], which may have diluted the survival benefit of pazopanib. Patients in the pazopanib arm were more likely to experience at least one on-therapy adverse event (AE) (99% versus 89%) and at least one serious AE (SAE) (41% versus 24%) compared with patients in the placebo arm [13]. The most frequently reported on-treatment AEs in the pazopanib group included fatigue, diarrhea, nausea, decreased weight, hypertension, and decreased appetite.

The primary objective of this study was to evaluate the cost-effectiveness of pazopanib versus best supportive care (BSC) from a UK healthcare system perspective when used in patients with aSTS who have received prior anthracycline-based chemotherapy.

## 2. Methods

### 2.1. Model Description

A multistate model was used to estimate expected PFS, OS, lifetime costs of treatment of aSTS, and quality-adjusted life-years (QALYs) in patients who had received prior treatment with chemotherapy and who were assumed to receive pazopanib, placebo, or chemotherapy. The model was developed in Microsoft Excel (Microsoft Corporation, Redmond, WA, USA).

Consistent with the modeling approach used in a multitude of other evaluations of treatments for advanced oncology indications [14–16], patients in the model were assumed to be in one of three mutually exclusive heath states at any given time: alive with no progression (PFS), alive with disease progression (postprogression survival [PPS]), or dead. The model was designed to permit two alternative approaches to estimate the proportion of patients in each health state over time. With the “partitioned-survival analysis” approach, survival distributions for PFS and OS were entered into the model, and the proportion of patients in the PPS state was calculated as the difference between OS and PFS. Alternatively, with the “Markov cohort analysis” approach, survival distributions for PFS and PPS were entered into the model, along with the estimated proportion of PFS events that are deaths. Transition probabilities were then derived from these inputs and combined to calculate the survival distribution for OS. With both approaches, expected costs and QALYs for each strategy were calculated as the product of the expected PFS and PPS and corresponding cost and utility value estimates for pre- and postprogression survival time, adjusted for “one-off” decrements in costs and quality of life associated with treatment initiation, AEs, progression, and death.

Expected lifetime outcomes and costs were evaluated over a 10-year timeframe, approximating a lifetime projection for patients with aSTS (i.e., virtually all patients were projected to be dead after 10 years). The model periodicity (i.e., the minimum duration of time a patient might remain in any disease state) was one week. Effectiveness measures were calculated on a discounted and undiscounted basis; costs were calculated on a discounted basis only. A 3.5% annual discount rate was employed beginning at the end of the first year of the model [17]. A UK healthcare system perspective was employed.

The primary analysis focused on direct comparisons of pazopanib versus BSC. In this analysis, data on PFS and OS for placebo patients from PALETTE were used without any adjustment of OS for the differential receipt of PTACT in the two groups. Utilization (and therefore costs) of PTACT was assumed to differ between groups as observed in PALETTE. For this analysis, the partitioned-survival analysis modeling approach was employed (i.e., the model took as inputs the distribution of OS rather than the distribution of PPS).

In a secondary analysis, pazopanib was compared with trabectedin (1.5 mg/m^2^), ifosfamide (3 g/m^2^), and gemcitabine plus docetaxel (900 mg/m^2^, 100 mg/m^2^). These therapies were the most relevant chemotherapy comparators in the UK based on treatment patterns reported in the SABINE study and consultation with clinical experts. In this analysis, a Markov cohort approach was employed, and it was assumed that PPS and PTACT utilization and costs would be the same for chemotherapy and pazopanib. Because PPS was assumed to be the same for pazopanib and chemotherapy, the difference between treatment strategies in mean OS was assumed to be equal to the difference in PFS (i.e., the benefits in PFS for pazopanib were assumed to translate directly to benefits of equal magnitude in OS). This approach was used because there was insufficient data from controlled trials to conduct robust adjusted indirect treatment comparisons of PFS and OS [18]. Accordingly, these comparisons were based on naive or unadjusted indirect comparisons. Because data on OS is more likely to be impacted by differences in patient populations and trial design than data on PFS, it was believed that the comparisons would be more reliable if based on PFS alone rather than PFS and OS. Also, there is no reason to believe that PPS would be different for patients receiving pazopanib versus chemotherapy.

### 2.2. Model Estimation

**Figure 1:**
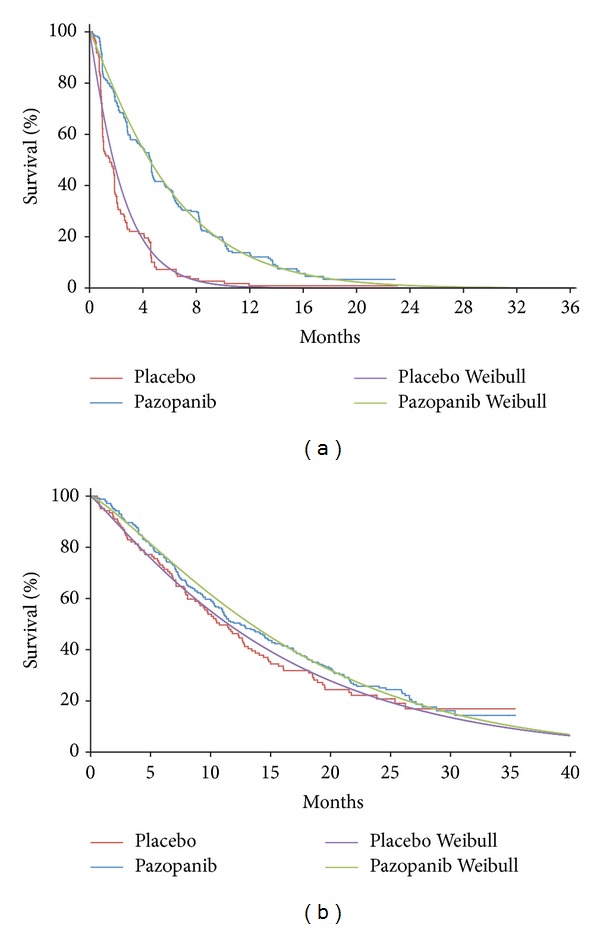
Weibull versus Kaplan-Meier survival distributions for (a) progression-free survival and (b) overall survival of pazopanib and placebo for patients in the PALETTE trial.

A summary of model inputs can be found in Table 1. PFS, OS, and PPS were estimated for pazopanib and placebo by fitting parametric survival functions to patient-level data from PALETTE using accelerated failure time regression (Figure 1) [19]. Investigator-assessed PFS (including clinical progression) was used as it was considered most likely to reflect PFS in clinical practice. OS was based on intent-to-treat analyses. Survival distributions for pazopanib and placebo were estimated independently. Exponential, Weibull, and log-logistic models were considered. Based on visual inspection and comparison of the restricted mean (i.e., area under the curve) for the empirical versus fitted distributions, the Weibull distribution provided the best fit for all distributions and was used in base-case analyses. Because the OS distribution for pazopanib derived from the PFS and PPS distributions in the Markov cohort analysis approach did not match the tail end of the empirical OS distribution well, the parameters of the PPS Weibull distribution were adjusted by calibrating the parameters of the distribution to minimize the differences between the model projections of expected OS and those obtained from the Weibull distribution fit to OS directly.

PFS for the other comparators was estimated by applying to the estimated PFS distribution for pazopanib estimates of the HRs for these treatments versus pazopanib using the formula

(1) $$\begin{array}{c} \text{PF}{\text{S}}_{\text{Comparator}}\left[t\right]=\text{PF}{\text{S}}_{\text{Pazopanib}}{\left[t\right]}^{{\text{HR}}_{\text{Comparator}\;\text{versus}\;\text{Pazopanib}}}. \end{array}$$

HRs for PFS for each comparator versus pazopanib were estimated using data from published studies of the comparators of interest that were identified based on a systematic review of the literature (Table 2). HRs were calculated by comparing the published Kaplan-Meier curves for PFS (or time to progression if PFS was unavailable) for the comparator(s) of interest in each study with the Kaplan-Meier PFS curve for pazopanib from PALETTE [20].

In PALETTE, the EuroQol 5-dimension (EQ-5D) health questionnaire was assessed only at baseline and at 4 weeks, whereas the European Organization for Research and Treatment of Cancer Quality of Life Questionnaire (EORTC QLQ-C30) was also assessed at 8 and 12 weeks [12, 13]. Therefore, a mapping algorithm was developed using data for the EQ-5D and QLQ-C30 at baseline and four weeks to predict EQ-5D utility values at 8 and 12 weeks given the responses to the EORTC QLQ-C30 at those later assessments [21]. The observed and mapped utility values were then combined to calculate mean utility values for each group for all pre- and postprogression assessments. Because the mean time from progression to postprogression utility assessment in PALETTE was only approximately one week in both groups, the mean differences in utility post- versus preprogression from PALETTE reflect declines in utility values immediately following progression only and do not reflect the declines in utility that would be expected over the entire postprogression period [22–25]. Accordingly, postprogression utility values for pazopanib and placebo were obtained by combining treatment group-specific estimates of the mean decrement in utility postprogression in PALETTE (reflecting the period immediately following progression) with an estimate of utility in the terminal phase of the disease. The latter was based on the estimated utility value for progressive disease from a vignettes study by Shingler and colleagues (mean [standard error], 0.263 [0.0231]) [26]. For the comparisons of pazopanib with chemotherapies, QALYs were adjusted for differences between treatments in the incidence of AEs using data on the incidence of AEs from PALETTE and the studies of chemotherapies noted above as well as disutility values for AEs that were obtained from published vignettes studies [26–28]. Utility values used in the model are reported in Table 3.

In the model, patients receiving pazopanib were assumed to incur the cost of a 28-day supply of pazopanib each 28-day cycle if they remained alive and progression-free. Any medication supplied but not taken was assumed to be discarded. Drug utilization was adjusted for dosage adjustment, early discontinuation, and interruptions. It was assumed that pazopanib would be provided to the UK National Health Service (NHS) at a 12.5% discount to the list price consistent with a published patient access scheme (PAS) agreement between the NHS and GlaxoSmithKline [29]. The cost of trabectedin medication was calculated assuming that the acquisition cost of trabectedin to the NHS would be capped at five cycles of treatment, consistent with a PAS agreement between the manufacturer of trabectedin (PharmaMar, Colmenar Viejo, Spain) and the NHS [30]. However, administration costs after five cycles were included in the cost estimate.

Expected costs of PTACT for each treatment group were calculated by combining Kaplan-Meier sample average estimates of the mean number of lines of PTACT received and treatment group-specific estimates of the distribution of PTACTs in PALETTE with corresponding estimates of the cost per course of each PTACT (for details, see Table 7) [31]. The mean duration of PTACT was assumed to be 4 months, which was an average of the mean PFS in the placebo and pazopanib arms in PALETTE (2.5 and 6 months, resp.). This value is similar to the mean time between lines of PTACT for patients who received more than one PTACT in PALETTE (4.5 months). Dosages for PTACTs were based on published studies and prescribing information.

Medication unit costs were obtained from the 63rd edition of the British National Formulary (details on drug and administration costs are listed in Table 4) [32]. Patients treated with ifosfamide were assumed to receive concomitant treatment with mesna to prevent urotoxicity. Patients receiving gemcitabine plus docetaxel were assumed to receive treatment with lenograstim to prevent neutropenic complications; those with prior pelvic irradiation were assumed to receive a 25% dose reduction of gemcitabine plus docetaxel [33]. Administration costs included the cost of dispensing pazopanib based on the hourly cost of a hospital pharmacist (as reported by Personal Social Services Research Unit) [34] and assumed each dispensation requires 15 minutes. Facility costs for administering trabectedin and ifosfamide were based on a weighted average of the outpatient and day case 2010/2011 NHS reference costs for HRG SB14Z, “delivery of complex chemotherapy (including prolonged infusional treatment at first attendance)” [35].

The costs of treatment of AEs were calculated by multiplying estimates of the incidence of AEs with estimates of the cost of treatment for each event (Table 8). Only grade 3–5 AEs for which the difference in incidence between pazopanib and placebo was ≥2% or that were considered by clinical experts to be of special interest were considered. Estimates of the incidence of AEs for pazopanib and placebo were from the PALETTE trial. Estimates of the incidence of AEs for chemotherapies were based on reported values in the literature. In the absence of a reported value, AE incidence was assumed to be equal to the AE incidence in the placebo arm of the PALETTE trial. All AEs were assumed to require one additional consultant visit, the cost of which was based on the NHS reference costs for HRG 800 “Clinical Oncology, Consultant Led: Follow up Attendance Non-Admitted Face to Face” [35]. The proportions of patients requiring hospitalization for each AE were estimated separately for pazopanib and placebo and were based on the proportions of events that were serious in each arm of PALETTE. Lacking similar data for trabectedin, ifosfamide, and gemcitabine plus docetaxel, the proportions of events resulting in hospitalization for these therapies were assumed to be the same as those for pazopanib. The costs of hospitalizations for AEs were based on 2010/11 NHS reference costs for nonelective inpatient stays for these events (long stay and short stay) [35] (Table 9).

Other STS-related direct medical costs were estimated based on a retrospective study of the cost of the management of metastatic STS in the UK by Judson and colleagues [36].

### 2.3. Sensitivity Analyses

Deterministic and probabilistic sensitivity analyses were undertaken to explore the effect of changing assumptions concerning key model parameter values on model results and cost-effectiveness acceptability curves generated [37, 38].

## 3. Results

### 3.1. Direct Comparison

In the base-case analyses (Table 5), pazopanib was estimated to increase QALYs by 0.128 and costs by *£*7,976 compared with placebo. The incremental cost-effectiveness ratio (ICER) of pazopanib versus placebo was estimated to be *£*62,162 per QALY gained.

**Figure 2:**
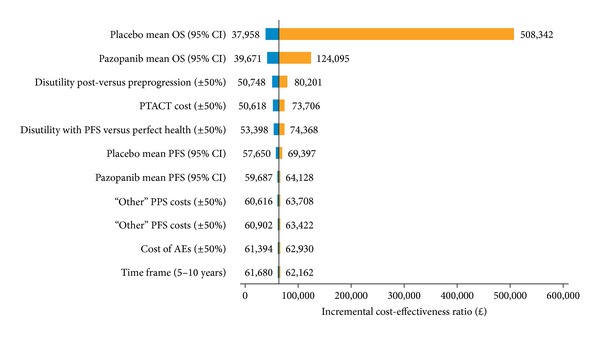
Tornado diagram showing result of deterministic sensitivity analysis for the direct comparison of cost-effectiveness of pazopanib versus placebo. Parameters were varied by 0.5 or 1.5 and shown on either side of the graph. AE: adverse events; CI: confidence interval; OS: overall survival; PFS: progression-free survival; PPS: postprogression survival; PTACT: posttreatment anticancer therapy.

Results for the deterministic sensitivity analysis are presented in Figure 2. In this analysis, the most influential variables were mean OS in the placebo group (range: *£*37,958–*£*508,342) and mean OS in the pazopanib group (range: *£*39,671–*£*124,095). For most parameters, the ICER changed <30% with ±50% changes in the parameter value.

**Figure 3:**
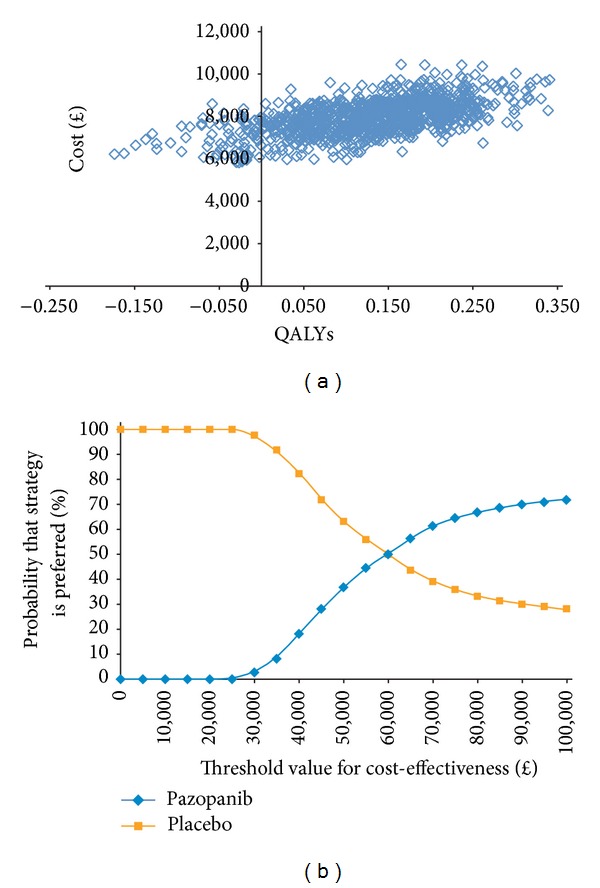
(a) Probabilistic sensitivity analyses for comparison of cost-effectiveness of pazopanib versus placebo. (b) Acceptability curve for comparison of cost-effectiveness of pazopanib versus placebo. QALYs: quality-adjusted life-years.

Probabilistic sensitivity analysis and acceptability curves for the comparison of pazopanib and placebo are presented in Figures 3(a) and 3(b), respectively. A high concentration of simulations was present in the north-eastern quadrant of the cost-effectiveness plane. Given a threshold value of *£*30,000 per QALY gained, there is an estimated 2.2% probability that pazopanib is preferred (i.e., considered to be cost-effective) versus placebo.

### 3.2. Indirect Comparisons

Results for comparisons of pazopanib versus ifosfamide, trabectedin, and gemcitabine plus docetaxel are summarized in Table 6. Pazopanib was estimated to gain 0.040, 0.029, and 0.001 QALYs versus ifosfamide, trabectedin, and gemcitabine plus docetaxel, respectively. Pazopanib was less costly than ifosfamide (*£*3,957 savings), trabectedin (*£*6,729 savings), and gemcitabine plus docetaxel (*£*2,692 savings). These results suggest pazopanib is dominant (i.e., lower costs and greater QALYs) compared with each comparator. In probabilistic sensitivity analyses, there was an estimated 100% probability that pazopanib is cost-effective versus ifosfamide or trabectedin and 98% probability versus gemcitabine plus docetaxel.

## 4. Discussion

This study was a cost-effectiveness evaluation of pazopanib in the treatment of patients with aSTS who have received prior chemotherapy from the perspective of the UK healthcare system. The primary analysis focused on a comparison of pazopanib and placebo. Based on cost-effectiveness criteria used by the National Institute for Health and Care Excellence (NICE), the results of our analysis suggest pazopanib is not cost-effective compared with placebo or BSC in these patients from a UK healthcare system perspective [39]. However, pazopanib might be cost-effective compared with trabectedin, ifosfamide, or gemcitabine when used in combination with docetaxel.

There is a dearth of information regarding the cost-effectiveness of other systemic agents to treat aSTS in the UK. Soini and colleagues [40] evaluated the cost-effectiveness of trabectedin versus end-stage treatment in Finland and reported ICERs ranging from €42,633 to €47,735/QALY depending on the utility values used in the model. A NICE evaluation of trabectedin versus end-stage treatment based on the same model reported an ICER of *£*56,985/QALY [41]. However, criticism of this model [42] has suggested that the incremental mean survival benefit of 21.1  months for trabectedin versus 7.2  months for end-stage treatment is clinically implausible and might be overestimated.

In the PALETTE study, there was a 3-month gain in PFS for pazopanib compared with BSC [12]. However, an imbalance in the use of PTACT between the two treatment arms may have affected the translation of the PFS benefit to OS. Of the 94% of patients who were off-protocol at the data cutoff date, 62% of patients in the placebo group and 45% of patients in the pazopanib group received additional chemotherapy, and 14% of patients in the placebo group versus 10% in the pazopanib group received targeted therapies. Given limitations in the data available from the PALETTE trial, it was infeasible to reliably adjust for differences in PTACT use. Therefore, we used OS data as observed and included the estimated costs of PTACT in each group. While this approach is internally consistent with respect to estimates of effectiveness and costs, it may not be generalizable to other settings, where treatment with placebo followed by PTACT is not a widely used treatment strategy.

While the PALETTE trial provided relatively robust information on utility values during PFS for patients treated with pazopanib and BSC, there was relatively little information on utility values after disease progression. Data from the PALETTE trial were combined with data from a vignettes study to estimate utility values for the PPS state [13, 23]. The model was relatively sensitive to the assumed decrement in utility following progression. Although utilities from vignettes studies have been used in numerous prior cost-effectiveness analyses of oncology therapies, the validity of the values obtained from such studies has never been formally assessed.

It is debatable whether placebo represents an appropriate treatment strategy given that the majority of patients in PALETTE went on to receive other active therapies. We therefore conducted a secondary analysis in which we compared pazopanib with other widely used chemotherapies. In this analysis, pazopanib was estimated to be dominant (i.e., provides greater QALYs at a lower cost) compared with trabectedin, ifosfamide, and gemcitabine plus docetaxel. However, the estimated differences in PFS and OS between pazopanib and these treatments were small and based on an unadjusted or naive indirect treatment comparison. Given the small differences and inherent uncertainty associated with these comparisons, no firm conclusion can be drawn with respect to the relative cost-effectiveness of pazopanib versus these treatments.

There may be other important factors besides cost-effectiveness that should be considered in reimbursement decisions regarding pazopanib in this indication. In particular, STS is an ultra-rare, incurable disease with short life expectancy and for which existing therapies are inadequate. Pazopanib represents a novel therapeutic class for the treatment of this condition. As an oral therapy, pazopanib may be an option for patients who wish to receive treatment at home.

## 5. Conclusion

From a UK healthcare system perspective, taking into account the threshold of *£*30,000/QALY, pazopanib is not cost-effective compared with BSC (base-case); however, pazopanib might be cost-effective compared with trabectedin, ifosfamide, or gemcitabine when used in combination with docetaxel.

## Figures and Tables

**Table 1:** Survival distribution inputs.

	Pazopanib	Placebo	Reference
Weibull survival function parameters			
PFS (months)			
Lambda	0.1279	0.3714	PALETTE
Gamma	1.1252	1.0809	PALETTE
OS (months)			
Lambda	0.0282	0.0469	PALETTE
Gamma	1.2341	1.1027	PALETTE
PPS (months)			
Lambda	0.104	0.118	PALETTE
Gamma	0.902	0.898	PALETTE
Probability of death without disease progression (required for Markov cohort model)^a^	0.053	0	PALETTE
HR for PFS for pazopanib versus comparator			
Ifosfamide	0.91 (95% CI, 0.73–1.14)		Sharma et al. 2013 [18] van Oosterom et al. 2002 [43]
Trabectedin	0.90 (95% CI, 0.76–1.07)		Sharma et al. 2013 [18] Demetri et al. 2009 [44] Garcia-Carbonero et al. 2005 [45] le Cesne et al. 2005 [46] Yovine et al. 2004 [47]
Gemcitabine + docetaxel	0.99 (95% CI, 0.70–1.40)		Sharma et al. 2013 [18] Hensley et al. 2008 [48] Pautier et al. 2012 [33]

CI: confidence interval; HR: hazard ratio; OS: overall survival; PFS: progression-free survival; PPS: postprogression survival.

^a^Same value was assumed for chemotherapies in indirect comparison.

**Table 2:** Studies used in indirect treatment comparison of progression-free survival.

Study	Design	Treatment	Control	Number of patients		Population	Outcomes assessed		
				Treatment	Control		TTP	PFS	OS
van der Graaf et al. 2012 [12]	Phase III RCT	Pazopanib 800 mg 1q1d	Placebo	246	123	Intermediate-/high-grade malignant STS excluding GIST and adipocytic subtypes, WHO PS 0/1, ≤4 prior systemic therapies for advanced disease and ≤2 combination regimens, age 18–83 y	No	Yes IRC and INV	Yes

Demetri et al. 2009 [44]	Phase II RCT	Trabectedin 1.5 mg/m^2^ 1q3w 24 h	Trabectedin 0.58 mg/m^2^ 1q1w 3 h	136	134	Unresectable and/or metastatic liposarcoma or LMS, ECOG PS 0/1, ≤2 prior cytotoxic regimens, age 20–80 y	IRC and INV	IRC, but KM curve not reported	Yes

Garcia-Carbonero et al. 2005 [45]	Phase II nonrandomized trial	Trabectedin 1.5 mg/m^2^ 1q3w 24 h		36		Histologically confirmed recurrent or metastatic STS with disease progression despite prior CT with ≤2 prior regimens for advanced disease, ECOG PS ≤ 1, age ≥ 18 y	Yes	No	Yes

le Cesne et al. 2005 [46]	Phase II nonrandomized trial	Trabectedin 1.5 mg/m^2^ 1q3w 24 h		104		Bidimensionally measurable metastatic or unresectable locoregional recurrent histologically proven advanced STS, ≥1 prior AC-based CT (±ifosfamide), PS ≤ 2, age < 75 y	Yes	No	Yes

Yovine et al. 2004 [47]	Phase II nonrandomized trial	Trabectedin 1.5 mg/m^2^ 1q3w 24 h		54		Advanced or metastatic, histologically proven STS, previous treatment with at least one line of either single-agent or combination CT, WHO PS ≤ 1, age ≥ 18 y	Yes	No	Yes

van Oosterom et al. 2002 [43]	Phase II RCT	Ifosfamide 5 g/m^2^ × 1q3w	Ifosfamide 3 g/m^2^ × 3q3w	36	40	Locally recurrent STS not amenable to treatment or metastatic STS, WHO PS 0–2, age 22–75 y	Yes	No	Yes

Hensley et al. 2008 [48]	Phase II nonrandomized trial	Gemcitabine 900 mg/m^2^ 2q3w + DOC 100 mg/m^2^ 1q3w		48		Women with advanced or recurrent uterine LMS progressed after 1 cytotoxic regimen, no prior therapy with gemcitabine or DOC, GOG PS 0–2	Yes	No	Yes

Pautier et al. 2012 [33]	Phase II RCT	Gemcitabine 900 mg/m^2^ 2q3w + DOC 100 mg/m^2^ 1q3w	Gemcitabine 1,000 mg/m^2^ 3q4w	69	52	Metastatic or unresectable LMS, PS ≤ 2, ≥1 prior AC-based regimen, age 41–80 y	No	Yes	Yes

AC: anthracycline; CT: chemotherapy; DOC: docetaxel; ECOG: Eastern Cooperative Oncology Group; GIST: gastrointestinal stroma tumor; GOG: Gynecologic Oncology Group; INV: investigator; IRC: institutional review committee; KM: Kaplan-Meier; LMS: leiomyosarcoma; OS: overall survival; PFS: progression-free survival; PS: performance status; RCT: randomized clinical trial; STS: soft tissue sarcoma; TTP: time to progression; WHO: World Health Organization.

**Table 3:** Utility values used in the model.

	Mean (SE)	Source
*Utility values for PFS and PPS, mean (SE) *		
Preprogression		
Pazopanib	0.674 (0.015)	PALETTE
Placebo	0.678 (0.024)	PALETTE
Postprogression		
Pazopanib	0.568 (0.044)	PALETTE
Placebo	0.636 (0.040)	PALETTE
*Estimated disutility values for AEs (SE) *		
Alopecia	0.045 (0.015)	Nafees et al. 2008 [27]
Anemia/hemoglobin	0.119 (0.023)	Swinburn et al. 2010 [28]
Asthenia/fatigue	0.262 (0.027)	Shingler et al. 2013 [26]
Cardiovascular (cardiac toxicity/left ventricular dysfunction)	0.2 (0)	Assumption
Decreased appetite/anorexia/weight decreased	0.2 (0)	Assumption
Diarrhea	0.327 (0.028)	Shingler et al. 2013 [26]
Edema	0.2 (0)	Assumption
Febrile neutropenia	0.09 (0.016)	Nafees et al. 2008 [27]
Leucopoenia/neutropenia/neutrophils/thrombocytopenia/low platelets	0.09 (0.015)	Nafees et al. 2008 [27]
Liver toxicity (ALT/AST elevation)	0	Assumption
Hypertension	0.153 (0.024)	Swinburn et al. 2010 [28]
Myalgia/muscle pain/neurotoxicity/peripheral sensory neuropathy	0.236 (0.025)	Shingler et al. 2013 [26]
Nausea/vomiting	0.357 (0.026)	Shingler et al. 2013 [26]
Pulmonary (dyspnea/pleural effusion/pneumothorax/pulmonary toxicity)	0.242 (0.026)	Shingler et al. 2013 [26]

AEs: adverse events; ALT: alanine aminotransferase; AST: aspartate aminotransferase; HR: hazard ratio; OS: overall survival; PFS: progression-free survival; PPS: postprogression survival; SE: standard error.

**Table 4:** Cost estimates used in the model.

Estimate	Costs, *£*	Reference
Medications unit costs		
Pazopanib, 200 mg tablet (no discount)	18.68	British National Formulary [32]
Pazopanib, 200 mg tablet (with discount)	16.35	
Trabectedin (per mg)^a^	1,366	
Ifosfamide (per mg)	0.04	
Gemcitabine (per mg)	0.14	
Docetaxel (per mg)	5.14	
Lenograstim (per mg)	238	
Mesna (per mg)	0.010	

Total costs of poststudy chemotherapy		
Placebo	11,493	PALETTE, British National Formulary [32]

Pazopanib^b^	8,531	NHS 2010-2011 Reference Costs [35]

Administration/dispensing		
Pazopanib	12.00	NHS 2010-2011 Reference Costs [35]
Ifosfamide	331.49	
Trabectedin	331.49	
Gemcitabine plus docetaxel	204.68	

Management costs^c^		
Preprogression monthly cost	92	Judson et al. 2007 [36]
Postprogression monthly cost	185	

^a^Costs are capped after five cycles; ^b^poststudy costs are assumed to be the same for pazopanib, ifosfamide, trabectedin, and gemcitabine + docetaxel in the indirect comparison; ^c^costs are updated to 2010/11 prices; preprogression costs are assumed to be half of postprogression costs.

**Table 5:** Base-case results for direct comparison of cost-effectiveness of pazopanib versus placebo.

	Pazopanib	Placebo	Pazopanib versus placebo
Effectiveness (discounted)			
LYs	1.375	1.262	0.113
PFLYs	0.503	0.211	0.292
PPLYs	0.872	1.051	−0.179
QALYs	0.719	0.591	0.128
Costs (discounted), *£*			
Study medication	10,733	0	10,733
Administration	81	0	81
Adverse events	251	54	197
Other costs PFS	557	234	323
Other costs PPS	10,463	13,822	−3,359
Total	**22,086**	**14,110**	**7,976**

Cost per QALY gained			62,162

LYs: life-years; PFLYs: progression-free life-years; PFS: progression-free survival; PPLYs: postprogression life-years; PPS: postprogression survival; QALYs: quality-adjusted life-years.

**Table 6:** Base-case results for pairwise indirect comparisons versus pazopanib (PPS-based analysis).

	Pazopanib^a^	Trabectedin	Ifosfamide	Gemcitabine + docetaxel	Pazopanib versus trabectedin	Pazopanib versus ifosfamide	Pazopanib versus gemcitabine + docetaxel
Effectiveness (discounted)							
LYs	1.375	1.334	1.336	1.373	0.041	0.039	0.003
PFLYs	0.503	0.461	0.463	0.500	0.042	0.040	0.003
PPLYs	0.872	0.874	0.873	0.872	−0.001	−0.001	0.000
QALYs	0.692	0.663	0.652	0.691	0.029	0.040	0.001
Costs (discounted), *£*							
Study medication	10,733	11,699	5,675	9,769	−965	5,059	964
Administration	81	2,491	6,516	2,707	−2,411	−6,435	−2,627
Adverse events	653	3,481	2,706	1,685	−2,828	−2,053	−1,033
Other costs PFS	557	1,080	1,082	554	−522	−525	3
Other costs PPS	10,464	10,466	10,466	10,464	−3	−3	0
Total	**22,488**	**29,217**	**26,445**	**25,180**	**−6,729**	**−3,957**	**−2,692**

Cost per QALY gained					Dominant	Dominant	Dominant

LYs: life-years; PFLYs: progression-free life-years; PFS: progression-free survival; PPLYs: postprogression life-years; PPS: postprogression survival; QALYs: quality-adjusted life-years.

^a^Because a Markov methodology was used for the indirect comparison, the effectiveness and cost results are similar but not identical to the direct comparison with placebo.

**Table 7:** Calculation of costs of posttreatment anticancer therapy.

Treatment	Medication cost^a^						Administration costs^b^			
	Mg per tab or vial	Cost per tab or vial, *£*	Cost per mg, *£*	Cost per administration, *£*	Expected days of use per course	Cost per course of therapy, *£*	Cost per administration, *£*	Expected days of use per course	Cost per course of therapy, *£*	Total cost per course of therapy, *£*
Cyclophosphamide	500	6	0	30	6.14	187	330	6.14	2,027	2,214
Dacarbazine	1,000	32	0	68	3.35	229	205	3.35	686	915
Doxorubicin	200	275	1	181	2.85	515	205	2.85	583	1,098
Etoposide	100	12	0	13	30.68	400	205	30.68	6,280	6,681
Etoposide + ifosfamide (+mesna)										
Etoposide	100	12	0	13	26.30	343	205	26.30	5,383	—
Ifosfamide	1,000	44	0	217	15.78	3,430	302	15.78	1,539	—
Mesna	400	4	0	46	15.78	725	—	15.78	—	—
Total	—	—	—	—	—	**4,499**	—	—	**6,922**	**11,421**
Gemcitabine	1,500	214	0.14	197	13.57	2,674	205	13.57	2,778	5,451
Gemcitabine + docetaxel (+lenograstim)										
Gemcitabine	1,500	214	0.14	185	8.90	1,643	205	8.90	1,821	—
Docetaxel	140	720	5.14	676	4.45	3,007	—	4.45	—	—
Lenograstim	0.263	62.54	238	64	31.14	1,988	0.57	31.14	18	—
Total	—	—	—	—	—	**6,639**	—	—	**1,839**	**8,478**
Ifosfamide (+mesna)										
Ifosfamide	1,000	44	0	217	15.78	3,430	302	15.78	4,769	—
Mesna	400	4	0	46	15.78	725	—	15.78	—	—
Total	—	—	—	—	—	**4,156**	—	—	**4,769**	**8,924**
Sorafenib	200	27	0	71	114.37	8,096	12	4.08	49	8,145
Sunitinib	12.5	28	2	99	65.33	6,489	12	1.56	19	6,508
Temozolomide	250	136	0.54304	109	23.67	2,570	12	0.85	10	2,580
Trabectedin	1	1,366	1,366	3,167	3.40	10,768	330	3.40	1,123	11,891

Tab: tablet; ^a^unit costs of medications based on British National Formulary [32, 35]; ^b^facility costs for administration of intravenous therapies are based on a weighted average of the outpatient, day case, and other 2010/2011 NHS Reference Costs for delivery of a chemotherapy cycle (SB12Z deliver simple parenteral chemotherapy at first attendance, and SB14Z deliver complex chemotherapy, including prolonged infusional treatment at first attendance for infusion of more than 24 hours) [35]. Assume dispensing costs of oral medications are based on the cost of a community pharmacist (15 minutes) [34].

**Table 8:** Random effects estimates of the probabilities of grade 3–5 AEs for each comparator based on Freeman-Tukey transformation (%)^a^.

Description of adverse event (% [SE])	Pazopanib	BSC	Trabectedin	Ifosfamide 3 g/m^2^	GEM/DOC
General					
Alopecia	0.1 (6.4)	0.2 (9.0)	0.5 (13.5)	64.6 (15.6)	0.5 (14.3)
Asthenia/fatigue	13.9 (6.4)	5.2 (9.0)	6.9 (15.5)	5.2 (9.0)	3.0 (14.3)
Hypertension	6.8 (6.4)	0.2 (9.0)	0.2 (9.0)	0.2 (9.0)	0.2 (9.0)
Myalgia/muscle pain	2.3 (6.4)	0.2 (9.0)	0.2 (9.0)	0.2 (9.0)	0.2 (9.0)
Neurotoxicity/peripheral sensory neuropathy	0.6 (6.4)	1.2 (9.0)	1.2 (9.0)	6.0 (15.6)	0.5 (14.3)
Edema	2.3 (6.4)	2.0 (9.0)	4.1 (16.7)	2.0 (9.0)	19.4 (14.3)
Liver toxicity					
ALT elevation	9.9 (6.5)	3.6 (9.0)	43.2 (12.5)	1.2 (9.0)	1.2 (9.0)
AST elevation	8.2 (6.5)	2.0 (9.0)	35.3 (8.2)	1.2 (9.0)	0.5 (14.3)
Cardiovascular					
Cardiac toxicity	0.1 (6.4)	0.2 (9.0)	0.2 (9.0)	0.2 (9.0)	0.2 (9.0)
Left ventricular dysfunction	1.9 (6.4)	0.2 (9.0)	0.2 (9.0)	0.2 (9.0)	0.2 (9.0)
Pulmonary					
Dyspnea	6.4 (6.4)	6.0 (9.0)	4.2 (8.7)	6.0 (9.0)	0.5 (14.3)
Pleural effusion	2.3 (6.4)	0.2 (9.0)	0.2 (8.7)	0.2 (9.0)	0.5 (14.3)
Pneumothorax	2.3 (6.4)	0.2 (9.0)	0.2 (9.0)	0.2 (9.0)	0.2 (9.0)
Pulmonary toxicity	0.1 (6.4)	0.2 (9.0)	0.2 (9.0)	0.2 (9.0)	9.2 (14.3)
Gastrointestinal					
Decreased appetite/anorexia	6.0 (6.4)	0.2 (9.0)	1.5 (7.3)	0.2 (9.0)	0.2 (9.0)
Diarrhea	4.8 (6.4)	1.2 (9.0)	0.9 (7.3)	1.2 (9.0)	1.2 (9.0)
Nausea/vomiting	6.8 (6.4)	2.8 (9.0)	13.5 (5.6)	18.3 (15.6)	3.0 (14.3)
Weight decreased	3.9 (6.4)	0.2 (9.0)	0.2 (9.0)	0.2 (9.0)	0.2 (9.0)
Hematologic abnormalities					
Febrile neutropenia	0.1 (6.4)	0.2 (9.0)	5.3 (14.5)	0.2 (9.0)	0.2 (9.0)
Anemia/hemoglobin	6.4 (6.4)	2.0 (9.0)	13.6 (9.9)	20.7 (15.6)	25.5 (14.3)
Leucopenia	1.4 (6.4)	0.2 (9.0)	43.2 (7.2)	64.6 (15.6)	23.5 (14.3)
Neutropenia/neutrophils	4.4 (6.4)	0.2 (9.0)	49.2 (8.7)	50.0 (15.6)	21.4 (14.3)
Thrombocytopenia/low platelets	3.9 (6.4)	0.2 (9.0)	15.7 (5.6)	15.8 (15.6)	39.8 (14.3)

AE: adverse event; ALT: alanine aminotransferase; AST: aspartate aminotransferase; BSC: best supportive care; GEM/DOC: gemcitabine + docetaxel; SE: standard error.

^a^SE on Freeman-Tukey transformation scale.

Note: if there is no information on AE then probability [SE] is assumed to be the same as placebo in PALETTE.

**Table 9:** Costs of adverse events.

AE	Probability that AE is serious		Cost per AE, *£*		Weighted average cost per AE, *£*	
	Pazopanib	Placebo	Nonserious	Serious	Pazopanib	Placebo
General						
Alopecia	100	0	0	0	0	0
Asthenia/fatigue	15	17	122	122	122	122
Hypertension	0	0	122	2,234	122	122
Myalgia/muscle pain	40	0	122	122	122	122
Neurotoxicity/peripheral sensory neuropathy	100	100	122	1,320	1,320	1,320
Edema	0	0	122	1,602	122	122
Liver toxicity						
ALT elevation	100	0	122	2,664	2,664	122
AST elevation	100	100	122	2,664	2,664	2,664
Cardiovascular						
Cardiac toxicity	100	0	122	3,843	3,843	122
Left ventricular dysfunction	100	0	122	3,843	3,843	122
Pulmonary						
Dyspnea	75	0	122	878	689	122
Pleural effusion	0	0	122	1,974	122	122
Pneumothorax	100	25	122	1,974	1,974	585
Pulmonary toxicity	100	0	122	1,713	1,713	122
Gastrointestinal						
Decreased appetite/anorexia	60	0	122	122	122	122
Diarrhea	0	0	122	1,171	122	122
Nausea/vomiting	7	0	122	1,171	195	122
Weight decreased	9	0	122	122	122	122
Hematologic abnormalities						
Febrile neutropenia	100	67	122	4,417	4,417	2,999
Anemia/hemoglobin	0	0	122	1,482	122	122
Leucopenia	0	0	122	4,417	122	122
Neutropenia/neutrophils	100	100	122	4,417	4,417	4,417
Thrombocytopenia/low platelets	50	50	122	2,051	1,086	1,086

AE: adverse event; ALT: alanine aminotransferase; AST: aspartate aminotransferase.

## Appendix

### Model Inputs and Parameters

See Tables 7, 8, and 9.
